# Interpersonal Victimization and Post-Traumatic Stress Among Transgender and Gender Expansive People: A Systematic Review

**DOI:** 10.3390/ijerph23050578

**Published:** 2026-04-29

**Authors:** Angie Wagner, Athena D. F. Sherman, Sarah Febres-Cordero, Sophie Grant, John Nemeth, Molly Szczech, Andrea Cimino, Carissa Lawrence, Sangmi Kim, Moriah Chedekel, Arlette Hernandez, Elijah Goldberg, Meredith Klepper, Pranav Gupta, Monique S. Balthazar

**Affiliations:** 1 School of Nursing, University of Minnesota, Weaver Densford Hall, 308 SE Harvard St., Minneapolis, MN 55455, USA; 2 Nell Hodgson Woodruff School of Nursing, Emory University, 1520 Clifton Rd., Atlanta, GA 30322, USA; ad-fsherman@emory.edu (A.D.F.S.); sfebre2@emory.edu (S.F.-C.); sophieggrant@gmail.com (S.G.); mszcze@emory.edu (M.S.); sangmi.kim@emory.edu (S.K.); mochedekel@gmail.com (M.C.); arlette.hernandez@emory.edu (A.H.); elijah.goldberg@emory.edu (E.G.); 3 Woodruff Health Sciences Center Library, Emory University, 1462 Clifton Road, Atlanta, GA 30322, USA; jnemeth@emory.edu; 4 Rogue Scholar, 2113 Lake Montebello Ter., Baltimore, MA 21218, USA; andrea@roguescholar.com; 5 Johns Hopkins School of Nursing, 525 N. Wolfe St., Baltimore, MD 21205, USA; clawre15@jhmi.edu (C.L.); mkleppe1@jhmi.edu (M.K.); 6 School of Medicine and Children’s Healthcare of Atlanta, Emory University, 2174 North Druid Hills Rd. NE, Atlanta, GA 30329, USA; pranav.gupta@emory.edu; 7 Ross and Carol Nese College of Nursing, The Pennsylvania State University, 201 Nursing Sciences Building, 235 E. College Ave., University Park, PA 16802, USA; msb6243@psu.edu

**Keywords:** mental health, PTSD, violence, survivors, transgender, victims

## Abstract

**Highlights:**

**Public health relevance—How does this work relate to a public health issue?**

- The mental health crisis is uniquely impacting transgender and gender-expansive (TGE) individuals, who experience disproportionately high rates of interpersonal violence across the lifespan, with correspondingly elevated rates of PTSD symptoms and diagnoses.

**Public health significance—Why is this work of significance to public health?**

- Mental health is inextricably linked with physical health, environment, economic status, and social wellbeing.
- Interpersonal violence is a significant factor in community health, and interventions to prevent and respond to violence can limit ongoing disease burden (in this case, PTSD).

**Public health implications—What are the key implications or messages for practitioners, policymakers and/or researchers in public health?**

- Anti-discrimination and anti-hate legislation, and community-campaigns promoting gender affirmation can mitigate interpersonal violence against TGE people.
- Universal screening for violence and PTSD exposure can help to identify survivors, and federally mandated gender-affirming and consent-focused sexual education is needed in public schools to prevent violence against TGE people.

**Abstract:**

Background: Transgender and gender expansive (TGE) people experience high rates of interpersonal victimization, which has been linked to high rates of post-traumatic stress disorder (PTSD, a highly disabling and under-studied mental illness among TGE people). This systematic review identifies, classifies, critically appraises, and synthesizes the peer-reviewed literature describing the association between interpersonal victimization and post-traumatic stress among TGE people. This review collates what is known about the associations between victimization and PTSD among TGE people and makes recommendations to guide future research and intervention development. Methods: Searches were conducted across five databases (PubMed, Embase, Web of Science, PsycInfo, and CINAHL) following PRISMA guidelines. Inclusion criteria were: English language; peer-reviewed original research; articles describing the association between victimization and PTSD among TGE youth or adults; reporting TGE-specific data. Exclusion criteria were: reviews, commentaries without original data, dissertations or theses, conference abstracts, animal studies, studies without TGE-specific findings, and case studies. Quality appraisal was completed for all studies, which included a discussion of bias. Data extraction was completed by two independent authors, and conflicts were resolved by a third. Data were stratified by gender identity, race or ethnicity, and type of violence for further synthesis. Results: 25 studies were evaluated for design, measure quality, and key findings. Findings were highly consistent across studies: multiple forms of interpersonal violence (e.g., childhood maltreatment, sexual violence, intimate partner violence, and transgender-specific victimization) were significantly associated with PTSD symptom severity or diagnosis across diverse identities and geographic contexts. All studies examining childhood sexual abuse reported significant associations with PTSD outcomes, highlighting early life as a critical period of vulnerability. Samples were disproportionately White and adult, with limited examination of intersectional experiences shaped by race, ethnicity, and socioeconomic status. Discussion: Interpersonal violence-related PTSD among TGE populations reflects a pervasive and systemic pattern of trauma rooted in structural discrimination rather than isolated individual risk. Addressing this inequity requires multilevel prevention and intervention strategies. Future research should prioritize longitudinal designs, culturally responsive measurement tools, and intersectional analyses to inform prevention, clinical care, and policy responses. The majority of studies were cross-sectional designs, so causality cannot be inferred. Additionally, the samples were disproportionately White and adult, which may bias the magnitude of associations reported and limit generalizability to racially and ethnically diverse TGE populations. Although many studies reported race and ethnicity descriptively, none disaggregated violence-related PTSD outcomes by racial or ethnic group within TGE samples, representing a critical limitation for intersectional analysis.

## 1. Introduction

Transgender and gender expansive (TGE) people face disproportionately high rates of victimization, contributing to adverse mental health outcomes. At least 25% of TGE people experience physical assault related to their gender identity, and 50–90% report verbal harassment or disrespect [1]. TGE individuals are 1.7 times more likely than cisgender peers to experience intimate partner violence (IPV), with a median lifetime prevalence of physical IPV of 37.5% [2]. Disproportionate exposure begins early, with higher rates of childhood abuse and school-based verbal victimization among transgender and nonbinary youth [3,4].

Post-traumatic stress disorder (PTSD, a highly disabling and under-studied mental illness affecting high rates of TGE people) is a psychiatric condition characterized by intrusive symptoms, avoidance, negative alterations in cognition and mood, and heightened arousal and reactivity following a traumatic event [5,6]. PTSD significantly impairs daily functioning across domains including self-care, interpersonal relationships, and occupational functioning [7], and disrupts social roles and relationships with partners, family members, and employers [8]. Violence exposure is strongly associated with post-traumatic stress, particularly when the violence is interpersonal or intentional [9,10,11]. Transgender veterans are 1.5–1.8 times more likely to have PTSD than cisgender veterans [12], a critical disparity because 20% of transgender people reported having served in the military compared to 10% of the general population according to the respondents of The National Transgender Discrimination Survey [13]. In a sample of 412 transgender adults, higher exposure to discrimination, childhood abuse, IPV, high visual gender nonconformity, and past-week depression were associated with higher PTSD severity [14].

Given the high prevalence of interpersonal victimization and PTSD among TGE people, and the dearth of research examining this relationship [5], this systematic review identifies, classifies, critically appraises, and synthesizes the peer-reviewed literature examining the association between victimization and PTSD among TGE people. Findings are used to summarize current evidence and inform recommendations for future research and intervention development.

### Theoretical Grounding

The Minority Stress Model posits that minoritized people experience external stressors (e.g., victimization) and internal stressors (e.g., internalized discrimination) related to minoritized identities and/or social positions, which comingle to impact mental health outcomes (e.g., PTSD) [15]. Additionally, intersectionality posits that one’s intersecting and overlapping social identities and positions are related to the oppression or privileges that they experience throughout their lives. Those social identities and positions are not additive when determining risk of oppression, but rather, they are multiplicative, with the highest exposure to oppression and adversity and worse outcomes seen among those with multiple marginalized social identities or positions [16]. This theoretical grounding guided variable selection, as well as aids us in better understanding findings and discussing implications of the current systematic review and state of the peer-reviewed literature.

## 2. Methods

### 2.1. Search Strategy and Selection of Articles

The search strategy for this systematic review was conducted according to PRISMA guidelines and developed with assistance from a Clinical Informationist with Emory University’s Woodruff Health Sciences Center (WHSC) Systematic Review research team who conducted database searches with the WHSC Library. PubMed’s Medical Subject Headings (MeSH) were used and translated across databases including PubMed, Embase, Web of Science, PsycInfo, and CINAHL. Search terms were identified in three categories, TGE population, mental health outcomes, and violence terms. TGE population terms included “male to female transgender,” female to male transgender,” “gender variance,” “androgyny,” “cross-dressing,” “transgender,” “transsexualism,” “transsexuality,” “trans man,” and “trans woman.” Mental health outcomes terms included “suicide,” “suicidal behavior,” “posttraumatic stress disorder,” “PTSD,” “posttraumatic stress,” “PTSS,” “self harm,” and “self injury.” Violence terms included “violence,” “victim,” “domestic abuse,” “domestic abuse,” “emotional abuse,” “assault,” “torture,” “bullying,” “polyvictimization.” Full search strings and strategy details are provided in the Supplemental Materials. Search protocol is available in PROSPERO (ID CRD42023398371).

The initial search focused on TGE individuals, violence, and any of the following mental health sequalae: depression, PTSD, and self-injurious thoughts and behaviors, yielding 3350 studies. Search results were uploaded into EndNote for duplication removal, then imported into Covidence for secondary duplicate removals. After removing duplicates, 2819 studies remained. Title and abstract screening yielded 185, undergoing full-text review, of which 25 studies met eligibility criteria (See Table 1). Screening was conducted by two independent reviewers, with conflicts resolved by a third reviewer.

During screening, the review scope was narrowed to PTSD outcomes due to feasibility considerations; PROSPERO was addended for this change in scope. Final inclusion criteria were: English language; peer-reviewed original research; articles describing the association between victimization and PTSD among TGE youth or adults; reporting TGE-specific data. Exclusion criteria included reviews, commentaries without original data, dissertations or theses, conference abstracts, animal studies, studies without TGE-specific findings, and case studies. No restrictions were placed on publication date or geographic location. There were no limits on dates or geographic locations. Searches were conducted on 23, 27, and 28 March 2023 with follow-up searches using the final criteria on 24 April 2024, and 1 April 2025. The 2024 and 2025 searches followed the same procedures related to TGE populations, PTSD, violence, and abuse.

### 2.2. Data Extraction, Analysis, and Synthesis

Each article underwent full-text review. Data to be extracted were determined by two authors who worked together. Data extraction was conducted independently by two authors using the Covidence Data Extraction tool, with conflicts resolved by a third reviewer. The extraction framework was developed collaboratively and finalized by one author. Extracted data included study location and population characteristics, study aims, violence exposure and protective factors, validated measures of violence and PTSD, and key findings related to the violence–PTSD association. Studies that did not report sought data were documented as “not reported.” Data were exported to Excel and stratified by gender identity, race or ethnicity, and type of violence for further synthesis (available upon request from the primary author).

### 2.3. Quality Appraisal and Thematic Analysis

Quality appraisal tools were selected by two authors and applied by one author. The CONSORT [17] checklist was used for longitudinal studies, the JBI Checklist for Analytical Cross-Sectional Studies for cross-sectional and mixed methods studies using quantitative data [18], and the McMaster Critical Review Form for Qualitative Studies for qualitative articles [19]. A 20% audit of quality appraisal was conducted by a second author for quality assurance. Discrepancies were resolved through a virtual meeting between the two reviewers; any remaining discrepancies were resolved by a third reviewer. Where sought information for quality appraisal was unclear, the authors reported they did not meet criteria. Study findings were synthesized through thematic analysis conducted by one author, with oversight from a second author.

## 3. Results

This review found that interpersonal victimization was consistently associated with increased PTSD diagnoses and symptom severity among TGE people of varying ages, gender identities, and geographic regions. Table 1 includes detailed descriptions of the identified studies, and the quality appraisal of studies can be found in Supplemental Materials. Most studies took place in the United States (U.S.; *n* = 20/25), with other locations including Australia (*n* = 3), and China (*n* = 1). Four articles presented U.S. aggregate data, with others presenting regional findings including San Francisco, New York City, Washington DC, Rhode Island, Massachusetts, and Maryland. Two articles in the U.S. presented findings from Southern states, one recruited participants from universities in Texas and Florida, and another recruited from a university in Charlotte, North Carolina. Thus, the review and synthesis of the articles provided below should be interpreted with the knowledge that pertinent articles in different languages may have been excluded. An array of gender identities were represented, with five articles based entirely on transgender women, one was entirely transmasculine people (assigned female at birth). Four had a majority, and one more had a plurality of gender-expensive people. Three had a majority transgender men. Two articles presented PTSD outcomes from aggregate transgender and gender expansive people. No studies were exclusively about nonbinary or gender expansive people.

**Table 1:** Description of identified studies.

Authors, Reference	Sample Location	Study Design: Sampling (Data Collection Method)	Sample Characteristics: TGD N, Gender/s, Race/Ethnicity of TGD People
Arayasirikul et al., [20]	United States (San Francisco Bay Area)	Longitudinal cohort cross-sectional survey: respondent-driven sampling, e-referrals and online social network site outreach (survey method NR).	300 (100% TW; 12% AA, 0.3% AIN, 31% HL, 1.3% PINH, 35.7% WH)
Beckman et al., [21]	United States	Cross-sectional survey: convenience sampling, via outreach to listservs, online groups, and organizations that serve transgender veterans, and Facebook (online survey).	221, 38 experienced Military Sexual Assault (23.7% TM, 76.3% TW; 5.3% NW, 94.7% WH)
Garcia et al., [22]	United States (Midwest)	Cross-sectional survey: snowball sampling on social media through personal and trans-community-specific pages, and recruitment of psychology students at a public midwestern university (survey).	160 (1.3% AG, 0.6% BG, 1.3% GF, 6.9% ML, 12.5% NB, 0.6% Q, 16.3% T, 13.1% TM, 0.6% TS, 6.9% TW, 1.9% Could not define, 38.1% Chose not to answer; 31.9% AA, 1.9% AF, 3.8% AIN, 6.9% AS, 20% LA or SPA, 40.6% WH, 2.5% Chose not to answer)
Grocott et al., [23]	United States	Cross-sectional survey: recruited through Amazon’s Mechanical Turk (online study).	191 (2.1% NB, 0.5% GL, 0.5% GNC, 11.0% GQ, 5.8% M, 24.6% TM, 44.5% TW, 9.9% W; 18.8% AA, 0.5% AS, 1.6% CH, 2.6% DTS, 0.5% GU, 4.2% I, 1.0% K, 7.9% MEX, 1.6 MU or BR, 1.0% PINH, 4.2% PR, 3.1% SP, 72.8% WH)
Hawkey et al., [24]	Australia	Qualitative: flyers and social media posts through organizations supporting LGBTQ communities, community migrant organizations, social media and snowballing (in-depth interviews and photovoice).	31 (29.0% F, 6.5% FA, 6.5% GF, 16.1 NB, 32.3% TW, 9.7% W; See article for racial breakdown)
Hughto et al., [25]	United States (Rhode Island and Massachusetts)	Cross-sectional survey: recruited through transgender-specific online and in-person venues (online and in-person computer survey).	545 (42.2% NB, 32.5% TM, 25.3% TW; 3.3% AA, 0.2% AI, 2.4 AS, 3.5% HS, 1.1% ME, 7.5% MU, 82% WH)
Kilimnik et al., [26]	United States (Florida and Texas)	Cross-sectional survey: recruited from two American universities (southeast and southwest), to take part in a larger study on alcohol and health-risk behaviors. Individuals were recruited by email invitations from registrar lists, flyers posted on campus and online, and through student research participant pools (survey).	106 (4.9% TGD *; 16.98% AA, 3.77% DTS, 17.92% HL, 6.60% MU, 60.38% WH)
Laughney et al., [27]	United States	Cross-sectional survey: Gallup screened for eligible transgender participants from April 2016–August 2016 to June 2017–December 2018 (secondary analysis of the U.S. Transgender Population Health Survey, data was obtained through the Inter-University Consortium for Political and Social Research online data archive).	274 (31.3% NB/T **, 30.9% TM, 37.8% TW; 9.5% AA, 15.7% L, 10.4% MU, 7.9% O, 56.5% WH)
Lindsay et al., [28]	United States	Cross-sectional survey: analysis of Veterans Health Administration medical records (review of medical records).	332 (23.5% TM, 76.5% TW; 10.5% AA, 1.2% AI, 1.2% AS, 0.6% DTS, 9.6% HS, 72.6% WH)
López et al., [29]	United States	Cross-sectional survey: online recruitment through social media (secondary analysis of a larger study on sexual violence disclosure in-person and online via #MeToo).	67 (14.4% NB *, Race/ethnicity NR)
Madzoska et al., [30]	Australia	Cross-sectional survey: random digit dialing of Australian Child Maltreatment Survey participants (retrospective interview administered using computer-assisted telephone interviewing).	126 (1.5% DGI *; Race/Ethnicity NR)
McDowell et al., [31]	United States (Boston, Massachusetts)	Cross-sectional survey: convenience sampling methods like recruitment flyers, medical provider and staff referrals, community outreach, social media, community listserv posts, and word-of-mouth referrals (in-person online survey, clinical visit, exit interview).	150 (76.7% BT/TMA, 23.3% NB/TMA; 2.7% AA, 6.0% AS, 9.3% HL, 16.0% MTO, 0.7% PINH, 74.7% WH)
McMillan et al., [32]	United States (Charlotte, North Carolina)	Cross-sectional survey: Enrolled undergraduate and graduate students were sent invitation emails from the Chancellor, Vice Chancellor of Student Affairs, and research staff over 6 weeks in Spring 2022 (20 min campus climate survey hosted on Qualtrics).	89 (5.9% TGD *, Race/ethnicity of TGD people NR)
Peitzmeier et al., [1]	United States (Boston, Massachusetts)	Cross-sectional survey: recruitment flyers aimed at Fenway Health patients were posted at clinical care sites; referrals from medical providers and clinical staff, and community recruitment, outreach to local organizations and venues frequented by members of the TM community; posts to social media, transgender websites, and email listservs; and word-of-mouth (in-person online survey, clinician-completed PAP specimen collection, post-interaction questionnaire, brief qualitative interview).	150 (78.0% BT/TMA, 22.0% NB/TMA; 75.2% WH, 24.8% POC)
Reisner et al., [14]	United States (Massachusetts)	Cross-sectional survey: transgender-specific online and in-person venues, transgender electronic listservs, emails, web postings on local community-based web sites, and social networking sites (majority completed online surveys, while some did the survey in-person via electronic tablets).	412 (59.7% BT, 62.6% FTM, MTF; NR; 2.9% AA, 9.0% HL, 4.4% MU, 2.9% O)
Sherman et al., [33]	United States (Baltimore, Maryland and Washington DC)	Secondary data analysis of cross-sectional survey: purposive sampling based on geographic location from the community and health care clinics (quantitative data collected from the multiphase study STROBE).	197 (100% TW; 61.9% AA, 9.1% AIN/I, 17.8% MU, 11.2% O)
Sherman et al., [34]	United States (Baltimore, Maryland and Washington DC)	Mixed Methods: word-of-mouth and flyers at transgender-serving community organizations, including organizations focused on transgender youth (semi-structured in-person interviews, community asset maps, trauma history timelines, and a tablet-based questionnaire).	151 quantitative (100% TW; 100% AA, with 7.3% HL); 19 qualitative (100% TW; 79% AA, 21.1% MX including AA)
Sherman et al., [35]	United States (Baltimore, Maryland and Washington DC)	Mixed Methods: distributing flyers to LGBTQ+ organizations and clinics, universities, and community colleges in both metro areas; LGBTQ+ and TGD group social media platforms; peer referral; and word-of-mouth (quantitative in-person researcher and computer-assisted survey; qualitative individual semi-structured interviews and a short survey).	19 qualitative (100% TW; 79% AA, 21.1% MX including AA)
Sherman et al., [36]	United States (with hubs in New York City, Boston, Atlanta, Baltimore, Miami, and Washington, DC)	Cross-sectional survey: LITE participants were recruited through dating apps, Google Ads, social media, peer referrals, in-person at transgender community events, and in clinical settings where gender-affirming and inclusive care was provided (data from American Cohort to Study HIV Acquisition among Transgender Women, the LITE Study).	1418 (100% TW/MTF; 19.7%, AA, 1.9% HI/AA, 10.2% HI/MX, 7.2% HI/WH, 13.6% MX, 1.1 U, 46.2% WH)
Strauss et al., [37]	Australia	Cross-sectional survey: social media (i.e., Twitter, Facebook and Tumblr), gender clinics, youth mental health services, support groups, parent and youth groups, and word-of-mouth (online survey).	859 (48.6% NB, 6.8% O, 29.7% TM, 15.5% TW; 3.7% ATS, 96.3% NATS)
Strauss et al., [38]	Australia	Cross-sectional survey: social media, gender clinics, youth mental health services, support groups, and word-of-mouth (online survey).	859 (48.6% NB, 6.8% O, 29.7% TM, 15.5% TW; 3.7% ATS, 96.3% NATS)
Stults et al., [39]	United States (New York City)	Cross-sectional survey: in-person events, social media posts (Facebook, Instagram, Twitter, Reddit), online dating apps (Tinder, Grindr), LGBTQ-related email listservs, and referrals from transgender health care providers and institutions, LGBTQ+ organizations, and previous participants (in-lab computer-based survey on Qualtrics).	200 (23.5% F, 34.5% GQ/GF/GNC/AG, 16% MA, 26.0% NB; 21.0% AA, 3.5% AS/API, 7.5% BR/MU, 28.5% LA, 3.0% O, 36.5% WH)
Suarez et al., [40]	United States	Secondary data analysis of mixed-methods study: print and online advertisements at Fenway Health clinic sites, electronic medical records, local social venues and events that attract transgender men, and participant and community referral (quantitative data collected from clinical trial on cervical cancer screening).	131 (100% TMA; 74.8% WH, 25.2% NW including MU)
Sun et al., [41]	China	Cross-sectional survey: universities’ staff members and targeted advertisements like WeChat student groups (online survey).	2352 (71.60% AFAB T/GNC, 28.40% AMAB T/GNC; 87.84% H, 12.16% O; See article for full ethnic breakdown)
Taber et al., [42]	United States (New York City)	Cross-sectional survey: in-person events, social media posts, online dating apps, LGBTQ+ health-related professional listservs; and referrals from transgender health care providers and institutions, LGBTQ+ organizations, and previous study participants (in-person survey).	200 (34.5% GQ/GF/GNC/AG/TS, 26.0% NB, 16.0% TM, 23.5% TW; 21.0% AA, 3.0% AIN/PINH, 3.5% AS, 7.5% BR/MU, 28.5% Latinx, 36.5% WH)

Note. GENDERS: AFAB: Assigned Female at Birth, AG: Agender, AMAB: Assigned Male at Birth, BG: Bigender, BT: Binary Transgender, DGI: Diverse Gender Identities, F: Female, FA: Fa’afafine, FTM: Female to Male Spectrum, GD: Gender Diverse, GF: Gender fluid, GL: Genderless, GNC: Gender Non-Conforming, GQ: Genderqueer, M: Man, MA: Male, MTF: Male to Female Spectrum, ML: Multiple Labels, NB: Nonbinary, O: Other, Q: Questioning, T: Transgender, TFE: Trans Feminine, TM: Transgender Man, TMA: Trans Masculine, TS: Two-Spirit, TW: Transgender Woman, W: Woman RACES or ETHNICITIES: AA: African American/Black, AF: African, AI: American Indian, AIN: American Indian or Alaskan Native, API: Asian Pacific Islander, AS: Asian, ATS: Aboriginal and/or Torres Strait Islander Descent, BR: Biracial, CH: Chinese, DTS: Declined to State, GU: Guamanian or Chamorro, H: Han, HI: Hispanic, HL: Hispanic or Latino, HS: Hispanic, I: Indigenous, K: Korean, LA: Latinx/Latino, LAC: Latin American, ME: Middle Eastern, MEX: Mexican/Mexican American/Chicano, MTO: More than one Race, MU: Multiracial, MX: Mixed Race, NA: North American, NATS: Not Aboriginal and/or Torres Strait Islander Descent, NW: Non White, O: Other, PINH: Pacific Islander/Native Hawaiian, POC: Person of Color, PR: Puerto Rican, SPA: Spanish, SP: Spanish/Hispanic/Latinx, U: Unknown, WH: White ADDITIONAL ABBREVIATIONS: PTSD: Post-traumatic Stress Disorder, IPV: Intimate Partner Violence, LGBTQ+: Lesbian, Gay, Bisexual, Transgender, Queer. * article presented data on TGD and cisgender people, only TGD data is presented here. ** Only nonbinary people who identified as transgender were included, nonbinary people who did not identify as transgender were excluded.

Across the included studies, samples were predominately white. Nine articles reported majority white participants, while five included racially diverse samples. Two articles included exclusively Black participants, and one had a majority Black sample. One study from China reported a majority Han sample. Two Australian studies reported samples predominately composed of participants who did not identify as Aboriginal and/or Torres Strait Islander descent. Although many studies reported participants’ race and ethnicity, none disaggregated rates of interpersonal violence-related PTSD by racial or ethnic group. Studies that included both cisgender and transgender participants often reported aggregate racial distributions without specifying the racial composition of TGE participants. Six studies reported PTSD prevalence stratified by race but did not explicitly assess whether participants had experienced violence.

The studies varied in design and methodology. Most were cross-sectional surveys (*n* = 19), with secondary data analysis of cross-sectional surveys (*n* = 2), two mixed methods studies, one longitudinal cohort cross-sectional survey, and one qualitative study. There were no randomized control trials. This reliance on cross-sectional surveys limits the ability to infer causality between interpersonal victimization and PTSD. Data collection methods were primarily survey-based (*n* = 22), followed by qualitative interviews (*n* = 6), clinical visits (*n* = 3), exit questionnaires (*n* = 3), secondary quantitative datasets (*n* = 2), review of medical records (*n* = 1), community asset mapping (*n* = 1), trauma history timelines (*n* = 1), and photovoice (*n* = 1). Sampling strategies varied and included respondent-driven sampling, snowballing, online recruitment, outreach through LGBTQ+ organizations and community spaces, recruitment via healthcare settings and provider referrals.

As shown in Table 2, the included studies assessed multiple forms of interpersonal violence, most frequently sexual violence, followed by physical violence, IPV, and polyvictimization. Less commonly examined exposures included transgender-specific IPV, psychological violence, military sexual violence, and threats of interpersonal violence. Several forms of victimization—child maltreatment, child sexual abuse, general interpersonal violence, transphobic childhood abuse, school bullying, technology-facilitated abuse, peer victimization, and internet-based bullying—were each addressed in a small number of studies. Studies varied considerably in how interpersonal victimization was measured. While several used established instruments (e.g., Identity Abuse Scale, Transgender-IPV Scale, Childhood Trauma Questionnaire, Sexual Experiences Survey–Short Form Victimization), others relied on adapted versions of existing measures without reporting reliability or validity. Some studies did not specify a formal measure and instead assessed victimization using binary yes/no items. Notably, among the instruments used, only the Transgender-IPV (T-IPV) Scale was developed and validated specifically within a transgender and gender expansive (TGE) population, demonstrating the need for more validated violence tools in this population.

Table 3 summarizes the measures used to assess PTSD diagnosis or symptoms, including measure descriptions, and reported reliability and/or validity. Most studies used the Primary Care PTSD Screen (*n* = 10), with one using the Primary Care PTSD Screen for DSM-5, or variants of the PTSD Checklist (PCL; *n* = 7), including Abbreviated PTSD Checklist (PCL-2), PTSD Checklist for DSM-5 (PLC-5), PCL-6, and the PLC-8. Other approaches included the Chinese Version of the Trauma Screening Questionnaire (*n* = 1), self-reported diagnosis by a provider (*n* = 2), diagnosis in a medical record (*n* = 1), qualitative interview (*n* = 1), the Mini-International Neuropsychiatric Interview Version 7.0.2 (MINI; *n* = 1), and Sexual Abuse Trauma Index (SATI) subscale from the Trauma Symptom Checklist-40 (*n* = 1). Most measures were self-reported, which limits the ability to validate this data, and only one measure was created and validated specifically with TGE people.

Table 4 presents key findings from the articles. Five studies exclusively sampled transgender women; of those, all reported significant positive correlations between interpersonal violence exposure and PTSD with no notable effects. Suarez et al. [40] exclusively sampled transmasculine people and found PTSD symptom severity was significantly associated with psychological abuse, physical abuse, sexual abuse, and neglect. The additional three articles with predominately transmasculine samples all reported positive correlations between interpersonal violence exposure and PTSD symptom severity. López et al. [29] and Madzoska et al. [30] focused exclusively on nonbinary people, and both reported significantly higher rates of PTSD compared to cisgender people. An additional nine studies with a majority of nonbinary people, all reported significant associations between interpersonal violence exposure and PTSD symptom severity or diagnosis. Of note, McMillan et al. [34] reported significantly higher post-traumatic stress related to technology-facilitated abuse (TFA) among TGD participants, than cisgender men (*p* = 0.034), when controlling for the number of TFA experiences.

Strength of association varied among the studies (detailed in Table 4), potentially influenced by the high variance in type of measures used, analyses conducted, and sample characteristics. However, herein, types of interpersonal violence exposure and associations with PTSD symptoms are synthesized to the extent possible without overstating the strength of the evidence.

### 3.1. Transgender Specific-Intimate Partner Violence and Identity Abuse

IPV against transgender people can take distinct forms that leverage transphobia as a mechanism of control. The T-IPV scale, developed by Peitzmeier et al. [1], assesses four manifestations of transgender-specific IPV: coercive control over gender transition or presentation, emphasizing the perceived undesirability of transgender people as intimate partners, blackmail through outing, and sabotage of gender transition.

In contrast, identity abuse (IA) measures IPV experiences related to gender identity and/or sexual orientation and captures homophobic, biphobic, and transphobic abuse [39]. The IA Scale assesses behaviors such as threatening to “out” someone, forcing unwanted public affection, weaponizing sexual or gender identity, questioning the legitimacy of someone’s identity, asserting someone deserves abuse due to their identity, using identity-based slurs, and restricting access to LGBTQ community support [43]. Three articles reported significant associations between PTSD symptom severity and T-IPV exposure. IA was also significantly associated with increased PTSD symptom severity in two studies. Strengths of association are compared by correlation coefficients [44]; articles reporting findings on polyvictimization, T-IPV, and SA had moderate strength. However, another study reporting significant associations between polyvictimization and lifetime PTSD score has only a weak association [33], indicating that violence type is likely not related to strength of association. Cohen’s d was used by Taber et al. [42], and found a medium effect of IA on PTSD scores, and a small effect of T-IPV on PTSD scores. Consistent with this, in Taber et al.’s [42] study, IA was directly associated with PTSD symptom severity, while T-IPV was not.

### 3.2. Child Maltreatment

Five studies examined childhood maltreatment and all reported significant associations between abuse and PTSD symptom severity/diagnosis. Childhood physical and sexual abuse were consistently associated with greater PTSD symptom severity, including when abuse extended into adulthood in Hughto et al.’s study [25]. However, in that study, the associations between adult abuse and PTSD symptoms were greater than associations between childhood abuse and PTSD symptoms [25]. Arayasirikul et al. [20] found transphobic verbal abuse in childhood was significantly associated with PTSD symptom severity. Among transmasculine adults in Suarez et al. [40], childhood psychological, physical, and sexual abuse, and neglect were each significantly associated with increased PTSD symptom severity. In Strauss et al.’s study [38] of 14–25-year-olds, lifetime PTSD diagnosis was associated with multiple forms of familial and extrafamilial abuse, and IPV. Two additional studies reported significant associations between PTSD symptom severity and peer bullying or assault. Laughney et al. [27] reported significantly higher PTSD symptom severity among adult transgender individuals with histories of childhood sexual abuse (CSA), a finding echoed by Garcia et al. [22], who observed a significant association between CSA and PTSD symptom severity among adult survivors.

### 3.3. Sexual Assault

Several studies examined sexual assault (SA) in relation to PTSD outcomes. Grocott et al. [23] and Reisner et al. [14] reported a significant positive correlation between SA exposure and PTSD. Conversely, Kilimnik et al. [26] reported participants who experienced SA had fewer post-traumatic stress symptoms than those who did not, in models that included drinking to cope and average alcohol consumption. Similarly, Grocott et al. [23] found social support from partners, family, or friends did not significantly moderate the relationship between sexual assault and SATI scores. Many transgender women qualitatively interviewed in Hawkey et al.’s study [24] described distress and PTSD following SA, particularly when perpetrated by a stranger and emphasized the psychological burden on anticipating sexual violence. Finally, two studies examined military sexual assault (MSA), with majority transgender women samples. Both reported significant positive associations between MSA and PTSD outcomes, including symptom severity [21] and diagnosis [28]. Lopez et al. [29] reported greater PTSD symptoms among transgender men who experienced MSA, than transgender women who experienced MSA.

## 4. Discussion

This integrative review synthesized evidence from 25 studies examining the association between interpersonal victimization and post-traumatic stress among TGE populations. Although this review was limited to articles available in the English language, the findings consistently demonstrate that TGE individuals experience disproportionately high rates of interpersonal violence across the lifespan, with correspondingly elevated rates of PTSD symptoms and diagnoses. Although the majority of studies were based on cross-sectional designs (thus, causality cannot be inferred), the evidence supports what TGE communities have long articulated: they face extraordinary rates of violence and its psychological consequences on a global scale.

Across somewhat diverse samples and settings, multiple forms of interpersonal violence were consistently associated with PTSD, reflecting a pervasive pattern of victimization rather than isolated or context-specific risk. However, of note, the majority of the studies had majority white adult transfeminine samples, which may bias the magnitude of associations reported, limit generalizability to racially and ethnically diverse TGE populations, and reduce our ability to better understand childhood experiences. Additionally, as 20/25 included studies are from the U.S., and these findings may be limited in their applicability to other geographic regions. Nonetheless, the largely consistent association between CSA and PTSD across the few studies which examined childhood experiences support the cumulative and multifaceted nature of trauma exposure in TGE populations and highlights early life as a potential critical period for prevention and intervention.

Measurement variability and limitations further constrain interpretation and synthesis across studies, reducing comparability across studies, the robustness of findings, and the kinds of claims that can be made. The limited availability of measures developed specifically with TGE populations also stands out as a structural gap in the research landscape. Across included studies, only one study utilized a violence measure that was validated in TGE populations to explicitly capture identity-specific victimization [1]. Cisgender-centric measures may fail to capture forms of transphobia and gender-based discrimination that escalate into victimization, leading to under-reporting and misclassification [45]. Similarly, although validated PTSD instruments were commonly used, many lacked cultural contextualization for identity-based trauma. Evidence suggests that the use of culturally appropriate probes can substantially alter PTSD prevalence estimates among TGE people; for example, one study found that PTSD Criterion A prevalence was 44% compared to 18% when culturally appropriate probes for discrimination were not incorporated into CAPS-5 administration [46]. Measures that lack cultural relevance may therefore contribute to both under-detection of trauma and further marginalization of TGE populations through mischaracterization of distress.

### 4.1. Implications

These findings point to the need for comprehensive, multilevel approaches that extend beyond individual survivors to address the social contexts that enable interpersonal violence against TGE people. Effective prevention requires shifting responsibility from individuals to the communities, institutions, and policies that target and fail to protect TGE people, especially TGE people of color [3]. Primary prevention strategies should include early identification of potential perpetrators of violence, implementation of anti-discrimination and anti-hate legislation, and community-level campaigns promoting gender affirmation [47,48]. Secondary prevention should incorporate universal screening for violence and PTSD exposure, and include education efforts including federally mandated, state-implemented gender-affirming sexual education in public schools [49,50]. Tertiary interventions must address both individual-level treatment needs through trauma-informed, gender-affirming approaches and structural barriers including discriminatory policing practices, housing discrimination, and healthcare access [3,51,52,53]. Future research should focus on the impact that these forms of structural violence have on experiences of violence and PTSD among TGE people.

Future research should prioritize sampling more racially, ethnically, and gender-diverse samples to better under the experience of the full community. Although many studies reported race and ethnicity descriptively, none disaggregated interpersonal violence-related PTSD outcomes by racial or ethnic group within TGE samples, representing a critical limitation for intersectional analysis. Future research is needed exploring interpersonal victimization experiences through an intersectional Multilevel Analysis of Individual Heterogeneity and Discriminatory Accuracy (MAIHDA) approach to better understand the implications of intersecting social identities [54]. Moreover, age variation in samples and longitudinal designs that explore how victimization and recovery trajectories unfold across the lifespan for TGE people are sorely needed. Such studies can inform the identification of critical intervention points to reduce long-term consequences of victimization and PTSD, including disability, morbidity, and premature mortality.

Additional research is needed to develop and validate culturally responsive violence and PTSD assessment tools for TGE populations, and that examine intersectional experiences across diverse racial, ethnic, and socioeconomic groups. The lack of research disaggregating interpersonal violence-related PTSD by race and ethnicity within TGE samples represents a critical gap, limiting understanding of how trauma occurs across intersecting systems of oppression and the compounding effects of racism and ethnic marginalization. Given the high prevalence of childhood maltreatment among TGE populations, additional research is needed to investigate intergenerational and epigenetic pathways and to evaluate interventions during family planning and conception.

### 4.2. Strengths and Limitations of the Current Systematic Review

This review has several strengths. It provides a comprehensive synthesis of peer-reviewed evidence examining the association between interpersonal victimization and post-traumatic stress among TGE people across diverse samples, gender identities, and geographic contexts. Despite heterogeneity in study design and measurement, findings were highly consistent and mirrored patterns observed in the cisgender literature on victimization and PTSD [23], often with substantially higher prevalence of both victimization and PTSD symptom severity among TGE populations [5]. The review also critically examined how interpersonal violence and PTSD were operationalized across studies, identifying key measurement gaps with implications for both prevalence estimates and clinical interpretation.

Some limitations of this review are of note. Gray literature was not included in this review, limiting the comprehensiveness of the review, leading to a potential for publication bias. The reviewed studies were limited to English-language literature, excluding potentially pertinent literature from other geographic regions.

## 5. Conclusions

Interpersonal violence-related post-traumatic stress among TGE people reflects a potentially pervasive and preventable public health inequity rooted in systemic discrimination and cumulative trauma. This review demonstrates consistent associations between multiple forms of victimization and PTSD across the TGE spectrum and confirms that addressing interpersonal violence and PTSD among TGE populations represents both a public health imperative and a human rights issue. Only through sustained commitment from researchers, clinicians, policymakers, and society—to improve the strength of the evidence and co-create/implement comprehensive, multilevel interventions—can we create environments where TGE people can live free from interpersonal violence and its traumatic aftermath.

## Figures and Tables

**Table 2:** Measurement and operationalization of violent victimization.

Types of Interpersonal Violence	Measures Used	Included Articles	Description of Scale	Reliability or Validity
Child maltreatment	Juvenile Victimization Questionnaire—R2: Adapted Version	Madzoska et al., [30]	Sixteen-item measure of 5 types of maltreatment: physical abuse (2 items), sexual abuse (4 questions), emotional abuse (3 items), neglect (3 items), and exposure to domestic violence (4 items).	NR
Child sexual abuse	ACEs: Sexual Abuse Questions	Laughney et al., [27]	Three questions asking if participants experienced sexual abuse by someone at least 5 years older than them, or an adult. Any yes answers constituted child sexual abuse.	NR
Childhood abuse (psychological, physical, sexual, neglect)	Adverse Childhood Events (ACEs)	Suarez et al., [40]	Ten-item measure to capture childhood trauma.	NR
Extrafamilial physical abuse, Familial physical abuse, Extrafamilial sexual abuse, Familial sexual abuse, Abuse in intimate relationship, Other familial abuse (including emotional or verbal abuse and neglect)	Did Not Report Specific Measure: Extrafamilial physical abuse, familial physical abuse, extrafamilial sexual abuse, familial sexual abuse, IPV, and familial emotional or verbal abuse and neglect	Strauss et al., [38]	Six free-text items capturing extrafamilial physical abuse, familial physical abuse, extrafamilial sexual abuse, familial sexual abuse, IPV, and other familial abuse (emotional or verbal abuse and neglect); coded as yes; no; partially, maybe, or sometimes; and unsure or No.	NR
IPV	The Composite Abuse Scale (Revised)-Short Form (CASR-SF)	Garcia et al., [22]	Fifteen-item measure, using gender-neutral language to assess for incidence of IPV, with 1 to 5 Likert scale to assess frequency of exposure.	Cronbach’s α = 0.85
IPV, Sexual IPV, Transgender-related IPV	Unnamed measure of physical IPV, sexual IPV, and transgender-related IPV	McDowell et al., [31]	Eleven-item measure (binary) developed by the researcher based on the following scales: Revised Conflict Tactics Scale (CTS2) and Transgender-IPV (T-IPV).	NR
Military sexual trauma/abuse/assault	MST was identified by a note in the medical record, as this is now a required screening for veterans presenting for treatment at the VHA	Lindsay et al. [28]	MST was identified by a note in the medical record, as this is now a required screening for veterans presenting for treatment at the VHA.	N/A
Physical, Sexual	Binary Yes/No from participant on childhood physical abuse, sexual abuse, and adult physical abuse or sexual abuse	Hughto et al., [25]	Participants were asked about experiences of abuse throughout the life course through measures previously utilized in transgender samples. Childhood physical abuse and sexual abuse were assessed before age 18. Physical and sexual abuse (partner and nonpartner) in adulthood (age 18 or older) were also assessed.	NR
Physical, Sexual, IPV	Binary Yes/No from participant reporting experience of Childhood abuse >15 y/o or IPV	Reisner et al., [14]	Two items asking, “Have you ever been slapped, punched, kicked, beaten up, or otherwise physically or sexually hurt by your spouse (or former spouse), a boyfriend/girlfriend, or some other intimate partner?” and “Were you ever physically or sexually abused as a child under age 15 years-old?”	NR
Physical, Sexual, Polyvictimization, Threats of violence	Polyvictimization Inventory (PVI)	Sherman et al, [33,34,35,36]	Fifteen-item measure ranging from 0 to 15, binary yes/no questions capturing sexual violence, physical violence, and threats of violence.	Cronbach’s α = 0.90 to 0.91
Physical, Verbal, Transphobic childhood adversity	Transphobic Childhood Physical Abuse	Arayasirikul et al., [20]	Composite measure of transphobic childhood adversity where none to one form of transphobic childhood verbal or physical abuse was categorized as low, and having both verbal and physical abuse was categorized as a high.	NR
Physical, Verbal, Transphobic childhood adversity	Transphobic Childhood Verbal Abuse	Arayasirikul et al., [20]	Composite measure of transphobic childhood adversity where none to one form of transphobic childhood verbal or physical abuse was categorized as low, and having both verbal and physical abuse was categorized as a high.	NR
Psychological, Peer Victimization, School Bullying	Did Not Report Specific Measure: Bulling and peer victimization	Strauss et al., [37]	Participants select all factors they have experienced from a list of negative experiences (including bullying).	NR
Sexual	Qualitative interview of participant experiences	Hawkey et al., [24]	In-depth interviews and photovoice conducted in English by a researcher who identified as a trans woman of color.	N/A
Sexual	Sexual Experiences Survey Short Form Victimization (SES-SFV)	Grocott et al., [23]; Kilimnik et al., [26]; López et al., [29]	Seven-item measure captures a range of unwanted sexual behaviors and assesses the tactics used by the perpetrator at the time of the assault.	Cronbach’s α = 0.89
Sexual, Military sexual trauma/abuse/assault	Adapted from the Sexual Experiences Survey	Beckman et al., [21]	Three-item measure of binary yes/no questions captures specific types of sexual assault (oral, vaginal, anal) that occurred during active military service.	NR
Sexual, Military sexual trauma/abuse/assault	Childhood Trauma Questionnaire	Beckman et al., [21]	Seventy-item measure captures physical and emotional abuse, emotional neglect, sexual abuse, and physical neglect; quantified on 5-point Likert-type scale according to frequency of experiences.	Cronbach’s α = 0.94
Technology-facilitated abuse	Technology-Facilitated Abuse in Relationships (TAR)	McMillan et al., [32]	Thirty-item measure, assessing for exposure to TFA.	Cronbach’s α = 0.90
Transgender-related IPV	Transgender-IPV (T-IPV)	Garcia et al., [22]; Peitzmeier et al., [1]; Stults et al., [39]; Taber et al., [42]	Four-item scale, measures TGD experience of IPV including coercive control of gender transition, emphasis of undesirability of TGD People, blackmail by outing, and sabotaging gender transition.	Cronbach’s α = 0.80 to 0.86; KR-20 score = 0.56
Transgender-related IPV, Identity abuse	Adapted from Conflict Tactics Scale (CTS)	Stults et al., [39]; Taber et al., [42]	Twelve items capturing experiences of psychological, physical, and sexual victimization and perpetration (6-items in the last year and 6-items lifetime).	NR
Transgender-related IPV, Identity abuse	Identity Abuse Scale (IA)	Stults et al., [39]; Taber et al., [42]	Seven-item scale that measures last year and lifetime IPV victimization with homophobic, biphobic, and/or transphobic content. IA measures IPV as it relates to one’s sexual identity and gender identity.	Lifetime α = 0.89; Past year α = 0.88
Verbal bullying, Physical violence, Sexual harassment, and Internet-based bullying and assault	Did Not Report Specific Measure: Peer Bullying	Sun et al., [41]	Binary yes/no of whether participants experienced peer bullying in the past year of the following types: verbal bullying, physical violence, sexual harassment, and internet-based bullying and assault.	NR

Note. IA: Identity Abuse, IPV: Intimate Partner Violence, MST: Military Sexual Trauma, N/A: Not applicable, NR: Not reported, TFA: Technology-Facilitated Abuse, TGD: Transgender and Gender-Diverse, T-IPV: Transgender-related Intimate Partner Violence, TMA: transmasculine, VHA: Veterans Health Administration.

**Table 3:** Measurement and operationalization of PTSD.

Measures Used	Included Article	Description of Scale	Reliability or Validity
17-item PTSD Checklist-Civilian	Beckman et al., [21]	Seventeen-item measure capturing the key symptoms of PTSD over the past month, with 5-point Likert scale ranging in frequency (“Not at all” to “Extremely”).	Cronbach’s α = 0.939
8-item Version of the PTSD Checklist 5 (PCL-8)	Kilimnik et al., [26]	Eight-item measure of PTSD symptoms, using a 5-point Likert scale measuring frequency of symptoms over the past month, symptoms statements cross all PTSD symptom clusters.	Cronbach’s α = 0.93
Abbreviated PTSD Checklist (PCL-2)	McMillan et al., [32]	Two-item measure assessing intrusive thoughts (e.g., repeated images of a past stressful experience) and distress associated with reminders of a past stressor since experiencing TFA. 5-point Likert scale ranging from 0 = not at all to 4 = extremely. The mean score was calculated between the two items.	Cronbach’s α = 0.95
Diagnosis by provider in VA medical record	Lindsay et al., [28]	Diagnosis of PTSD within the medical record.	N/A
Post traumatic Stress Disorder Checklist for DSM-5 (PCL-5)	Garcia et al., [22]	Twenty-item measure, with 5-point Likert scale the frequency of experiencing PTSD symptoms.	Cronbach’s α = 0.97
Post-traumatic Stress Disorder Checklist (PCL-6)	López et al., [29]; Stults et al., [39]; Taber et al., [42]	Six-item scale that evaluates experiences of PTSD over the past month, scored on a 5-point Likert scale ranging from 1 (not at all) to 5 (extremely), and a total score was summed with higher scores indicating greater severity of depression.	Cronbach’s α = 0.85 to 0.88
Primary Care PTSD Screen	Arayasirikul et al., [20]; Hughto et al., [25]; Laughney et al., [27]; McDowell et al., [31]; Peitzmeier et al., [1]; Reisner et al., [14]; Author’s Own [33]; Author’s Own [34]; Author’s Own, [35]; Suarez et al., [40]	Four-item measure of binary yes/no answers capturing reexperiencing, avoidance, numbing, and hyperarousal; a score of 3 or more is indicated for high PTSD risk.	Cronbach’s α = 0.78 to 0.87; Test–retest Pearson’s correlation coefficient = 0.83 (*p* < 0.001)
Primary Care PTSD Screen for DSM-5 (PC-PTSD-5)	Sherman et al., [36]	Five-item binary yes/no measure of lifetime PTSD symptom severity, with a score of 3 or more is indicative of a need for further assessment or intervention.	NR
Qualitative interview of participant experiences	Hawkey et al., [24]	In-depth interviews and photovoice conducted in English by a researcher who identified as a transgender woman of color.	N/A
Self-Reported Diagnosis by provider	Strauss et al., [37,38]	Participant Reported Binary Yes/No of PTSD Diagnosis from a Health Professional.	N/A
Sexual Abuse Trauma Index (SATI) subscale from the Trauma Symptom Checklist-40	Grocott et al., [23]	Seven-item subscale of the TSC-40 capturing a range of trauma symptoms participants rate the frequency each symptom over the past 2 months, answers fall on a 4-point Likert scale ranging from (“0 Never” to “3 Often”).	Cronbach’s α = 0.79
The Mini-International Neuropsychiatric Interview Version 7.0.2 (MINI)	Madzoska et al., [30]	A brief, structured diagnostic interview tool administered by trained interviewers, according to the DSM-5 criteria for GAD, PTSD (current), AUD (current), and MDD (lifetime).	NR
Trauma Screening Questionnaire (Chinese version)	Sun et al., [41]	Ten items designed to measure PTSD symptoms.	NR

Note. AUD: Alcohol Use Disorder, DSM-5 Diagnostic and Statistical Manual for Mental Disorders 5th Edition, GAD: Generalized Anxiety Disorder, MDD: Major Depressive Disorder, NR: Not reported, N/A: Not applicable, PTSD: Post-Traumatic Stress Disorder, TFA: Technology-facilitated abuse.

**Table 4:** Key findings.

Included Articles	Key Findings	Strength of Association
Arayasirikul et al., [20]	PTSD symptom severity was significantly associated with transphobic childhood verbal abuse (aOR = 2.06, 95% CI = 1.17–3.64, *p* < 0.05) when adjusted for age, race/ethnicity, educational level, monthly income, and housing situation growing up.	N/A
Beckman et al., [21]	Participants who experienced MSA (M = 53.25, SD = 18.44) demonstrated significantly higher severity of PTSD symptoms, t(210) = −3.13, *p* = 0.002, compared to the participants who did not.	Point-based specific to the measure used
Beckman et al., [21]	There was a significant positive correlation between MSA and PTSD scores (r = 0.21, *p* < 0.01).	Weak
Beckman et al., [21]	MSA was significantly associated with an (B = 10.18, SE = 3.42, *p* < 0.01) increase in PTSD symptom severity in a model accounting for gender identity, race, and age (df(4, 207), Model F = 4.87, *p* < 0.01, R2 = NR))	N/A
Garcia et al., [22]	T-IPV was significantly correlated with PTSD symptom severity (r = 0.45, *p* < 0.001).	Moderate
Garcia et al., [22]	IPV and PTSD were found to have a significant relationship (r = 0.38, *p* < 0.001). IPV was significantly associated with PTSD symptom severity when accounting for the interaction between IPV, transgender-specific structural social support, and living in one’s affirmed gender (b = 65.95, 95% CI = 20.31–111.59).	Weak
Garcia et al., [22]	IPV was significantly associated with PTSD symptom severity when accounting for the interaction between IPV, general structural social support, and living in one’s affirmed gender (b = 87.33, 95% CI = 31.85–142.82).	N/A
Garcia et al., [22]	IPV was not significantly associated with PTSD symptom severity when accounting for the interaction between IPV, transgender-specific perceived social support, and living in one’s affirmed gender (b = 44.18, 95% CI = −0.82–89.19).	N/A
Garcia et al., [22]	IPV was not significantly associated with PTSD symptom severity when accounting for the interaction between IPV, general perceived social support, and living in one’s affirmed gender (b = 50.33, 95% CI = −11.25–111.92).	N/A
Grocott et al., [23]	There was a significant positive correlation between sexual assault and PTSD scores (r = 0.55, *p* < 0.01).	Moderate
Grocott et al., [23]	In a series of models, sexual assault significantly predicted a 0.03 to 0.04 point increase (*p* < 0.001) in sexual assault trauma symptoms in models examining the effect modification of various forms of social support accounting for age, income, and symptoms of depression.	Point-based specific to the measure used
Hawkey et al., [24]	Many women reported psychological distress, including anxiety, depression and post-traumatic stress disorder as a direct result of sexual violence, particularly when perpetuated by a stranger. Constantly anticipating sexual violence was also positioned as having significant implications for women’s mental health.	N/A; Qualitative
Hughto et al., [25]	Childhood abuse was significantly associated with an increased severity of PTSD symptoms (OR = 2.55, 95% CI = 1.74–3.74, *p* < 0.0001).	N/A
Hughto et al., [25]	Childhood abuse was significantly associated with an increased severity of PTSD symptoms (OR = 1.92, 95% CI = 1.23–2.99, *p* < 0.004) adjusted for age, gender identity, race, education, income, adult abuse, and negative transgender-related media messages.	N/A
Hughto et al., [25]	Adult abuse was significantly associated with an increased severity of PTSD symptoms (OR = 3.24, 95% CI = 2.20–4.76, *p* < 0.0001).	N/A
Hughto et al., [25]	Adult abuse was significantly associated with an increased severity of PTSD symptoms (OR = 2.28, 95% CI = 1.48–3.51, *p* < 0.0002) adjusted for age, gender identity, race, education, income, childhood abuse, and negative transgender-related media messages.	N/A
Kilimnik et al., [26]	Sexual assault severity was negatively associated with PTSS (B = −0.866, SE = 0.177, *p* < 0.001) in pathway mediation models with drinking to cope and average number of drinks.	N/A
Laughney et al., [27]	Child sexual abuse was significantly associated with increased incidence of PTSD (OR = 3.6, *p* ≤ 0.001).	N/A
Laughney et al., [27]	Transgender adults who experienced CSA had a significantly higher incidence of PTSD compared to transgender adults who had not experienced CSA in models adjusting for substance use (aOR = 9.3, 95% CI = 3.9–22.5).	N/A
Lindsay et al., [28]	Transgender Men: There was a positive correlation between MSA and PTSD diagnosis (Estimate = 1.81, OR = 6.09, *p* < 0.05) controlling for race/ethnicity and age.	N/A
Lindsay et al., [28]	Transgender Women: There was a positive correlation between MSA and PTSD diagnosis (Estimate = 0.88, OR = 2.42, *p* < 0.05) controlling for race/ethnicity and age.	N/A
López et al., [29]	Among nonbinary survivors of sexual violence, rates of PTSD were higher [M = 3.38 (SD = 0.97), T = −4.28, *p* < 0.001, d = 0.94] than a group of cisgender and transgender women who were survivors of sexual violence [M = 2.88 (SD = 0.93), T = −4.28, *p* < 0.001, d = 0.94].	N/A
Madzoska et al., [30]	Among people with diverse gender identities who experienced child maltreatment, incidence of PTSD was 17.6 times higher than cisgender men with no child maltreatment, 95% CI = [7.8, 39.6], when adjusted for age.	N/A
Madzoska et al., [30]	Among people with diverse gender identities who experienced child maltreatment, incidence of PTSD was 9.5 times higher than cisgender men with no child maltreatment, 95% CI= [3.7, 24.2], when adjusted for age, financial hardship in childhood, current financial strain, and socioeconomic status.	N/A
McDowell et al., [31]	PTSD symptom severity was significantly associated with lifetime IPV (OR = 3.75; 95% CI = 1.72–8.17, *p* < 0.001).	N/A
McDowell et al., [31]	PTSD symptom severity was significantly associated with lifetime IPV (aOR = 3.08, 95% CI = 1.26–7.53, *p* < 0.01) controlling for everyday discrimination.	N/A
McMillan et al., [32]	TGD participants reported significantly higher TFA-related traumatic stress than cisgender men (B = 0.15, SE = 0.07, β = 0.05, *p* = 0.034), controlling for TFA experiences.	N/A
Peitzmeier et al., [1]	T-IPV is significantly associated with increasing PTSD symptom severity over the past 30 days (aOR = 2.23; *p* < 0.05), when adjusted for age, race, education, annual household income, gender identity, sexual orientation, and hormone use.	N/A
Reisner et al., [14]	Physical and/or sexual abuse <15 years of age is significantly positively correlated with PTSD symptoms (B = 0.29, 95% CI = 0.21–0.27, *p* < 0.0001) in models accounting for everyday experiences of discrimination, number of reasons for discrimination, IPV, depression, polydrug use, age, FTM, nonbinary identity, full-time social gender transition, medical gender affirmation, high visual gender nonconformity, people of color, income, educational attainment, unstably housed, sexual minority status, and online survey mode.	N/A
Reisner et al., [14]	IPV is significantly positively correlated with PTSD symptoms (B = 0.18, 95% CI = 0.10–0.26, *p* < 0.0001) in models accounting for everyday experiences of discrimination, number of reasons for discrimination, physical and/or sexual abuse <15 years of age, depression, polydrug use, age, FTM, nonbinary identity, full-time social gender transition, medical gender affirmation, high visual gender nonconformity, people of color, income, educational attainment, unstably housed, sexual minority status, and online survey mode.	N/A
Sherman et al., [33]	There is a positive correlation between polyvictimization and lifetime PTSD scores (r = 0.38, *p* < 0.01). Increased polyvictimization was significantly associated with increased PTSD symptom severity (B = 0.13, SE = 0.02, *p* < 0.0001), in a model accounting for age and city of residence [F(2, 193) = 12.78, *p* < 0.001; R2 = 0.153].	Weak
Sherman et al., [33]	There is a positive correlation between polyvictimization and PTSD (r = 0.38, *p* < 0.01, *n* = 197).	Weak
Sherman et al., [33]	There was a positive correlation between polyvictimization and PTSD symptom severity (r = 0.40, *p* < 0.01; two-tailed).	Moderate
Sherman et al., [33]	Increased polyvictimization was significantly associated with increased PTSD symptom severity (b = 0.3170, *p* < 0.001), in a step 1 model accounting for age and city of residence [F(3,147) = 6.6176, *p* < 0.001; R2 = 0.11].	N/A
Sherman et al., [33]	Step 2: Polyvictimization (b = 0.2622, *p* < 0.001) and barriers to healthcare access (b = 0.3357, *p* < 0.001) were positively associated with PTSD symptom severity, accounting for age and city of residence [F(4, 146) = 14.2746, *p* < 0.001; R2 = 0.281].	N/A
Sherman et al., [33]	Findings suggest that polyvictimization has a direct and indirect effect [Effect = 0.0338, 95% C.I. (0.0156, 0.0548)] on PTSD symptom severity when accounting for barriers to healthcare, age, and location of residence.	N/A
Sherman et al., [33]	Among the in-person sample, polyvictimization was significantly associated with increased PTSD severity while accounting for age, race, sampling zone, HIV, discrimination, food insecurity, social support, health insurance, homelessness, sex work, substance use, and employment (B = 0.112, 95% CI = 0.086–0.138, b = 0.291, *p* < 0.001).	N/A
Sherman et al., [33]	Among the online sample, polyvictimization was significantly associated with increased PTSD severity while accounting for age, race, discrimination, food insecurity, social support, health insurance, homelessness, sex work, substance use, and employment (B = 0.163, 95% CI = 0.125–0.202, b = 0.377, *p* < 0.001).	N/A
Strauss et al., [37]	Lifetime diagnosis of PTSD is significantly positively associated with bullying (OR = 1.904, 95% CI = 1.215–2.984, *p* < 0.005) when adjusted for sex assigned at birth and age.	N/A
Strauss et al., [38]	Lifetime diagnosis of PTSD is significantly positively associated with extrafamilial sexual abuse (OR = 3.970, 95% CI = 2.667–5.910, *p* < 0.001), extrafamilial physical abuse (OR = 4.096, 95% CI = 2.639–6.358, *p* < 0.001), abuse within an intimate relationship (OR = 1.971, 95% CI = 1.344–2.889, *p* = 0.001), familial sexual abuse (OR = 4.674, 95% CI = 2.554–8.554, *p* < 0.001), familial physical abuse (OR = 3.562, 95% CI = 2.417–5.247, *p* < 0.001), and other familial abuse (OR = 2.728, 95% CI = 1.826–4.077, *p* < 0.001) when adjusted for age and sex assigned at birth.	N/A
Stults et al., [39]	PSTD symptom severity was significantly correlated with lifetime psychological IPV (r = 0.20, *p* < 0.01), lifetime physical IPV (r = 0.20, *p* < 0.01), lifetime sexual IPV (r = 0.19, *p* < 0.01), lifetime T-IPV (r = 0.22, *p* < 0.01), lifetime IA (r = 0.27, *p* < 0.001), past-year psychological IPV (r = 0.18, *p* < 0.05), past-year physical IPV (r = 0.15, *p* < 0.05), past-year T-IPV (r = 0.24, *p* < 0.01), and past-year IA (r = 0.19; *p* < 0.01). PTSD symptom severity was not significantly correlated with past-year sexual IPV (r = 0.06, p NR).	Weak
Stults et al., [39]	Step 2: Lifetime IA was significantly associated with increased PTSD symptom severity (B = 3.18, SE = 1.20, *p* < 0.01) in models accounting for age, being born in the United States, generational status, race, gender identity, sexual identity, socioeconomic status, employment, education, and whether they are living as their affirmed gender.	N/A
Stults et al., [39]	Step 3: Lifetime IA was significantly associated with increased PTSD symptom severity (B = 3.48, SE = 1.35, *p* < 0.05) in models accounting for age, being born in the US, generational status, race, gender identity, sexual identity, socioeconomic status, employment, education, and whether they are living as their affirmed gender.	N/A
Stults et al., [39]	The step 2 and 3 model variances and significance were not reported.	N/A
Suarez et al., [40]	PTSD symptom severity was significantly positively associated with psychological abuse (chi-squared: 39.2, *p* < 0.05), physical abuse (chi-squared: 56, (*p* < 0.001), sexual abuse (chi-squared: 46, *p* < 0.01), and neglect abuse (chi-squared: 57.1, *p* < 0.01).	N/A
Sun et al., [41]	PTSD symptom severity was significantly positively associated with peer bullying and assault (aOR = 2.95, *p* < 0.001) adjusted for gender identity, age, family income, ethnicity, home residence, only-child status, and loneliness.	N/A
Taber et al., [42]	PTSD symptom severity was significantly positively associated with IA (Yes Mean = 13.38, SD = 6.22; No Mean = 9.83, SD = 6.40; *p* < 0.001; Cohen’s d = 0.56).	Medium Cohen’s d
Taber et al., [42]	The direct relationship between T-IPV and PTSD scores was not significant (PM = 1.45, SE = 0.88, 95% CI = −0.28−3.18).	No Association
Taber et al., [42]	PTSD symptom severity was significantly positively associated with T-IPV (Yes Mean = 13.93, SD = 6.67; No Mean = 10.88, S = 6.28; p < 0.002; Cohen’s d = 0.47).	Small Cohen’s d
Taber et al., [42]	The direct relationship between IA and PTSD was significant (PM = 2.00, SE = 0.86, 95% CI = 0.30−3.71).	N/A

B: Standardized beta, b: Unstandardized beta, BHI: barriers to healthcare, HIV: Human Immunodeficiency Virus, IA: identity abuse, IPV: intimate partner violence, MSA: military sexual assault, N/A: Not applicable, NR: Not Reported PTSD: Post-Traumatic Stress Disorder, PTSS: post-traumatic stress symptoms, SA: sexual assault, SATI: Sexual Abuse Trauma Index, T-IPV: transgender-related intimate partner violence, US: United States.

## Data Availability

No new data were created in this study. Compiled data is available by request from the corresponding author.

## Supplementary Materials

The following supporting information can be downloaded at: https://www.mdpi.com/article/10.3390/ijerph23050578/s1, Figure S1: The Preferred Reporting Items for Systematic Reviews and Meta-Analyses flow diagram.; Table S1: Quality Appraisal of Included Articles; File S1. PRISMA Checklist.
