# Supplementary material for: Interpersonal Victimization and Post-Traumatic Stress Among Transgender and Gender Expansive People: A Systematic Review
Source: Int J Environ Res Public Health. 2026 Apr 29;23(5):578. doi: 10.3390/ijerph23050578 (PMC13205732; doi:10.3390/ijerph23050578)
Supplement: Supplementary file 1 [file ijerph-23-00578-s001.zip › ijerph-4244164-supplementary.pdf]

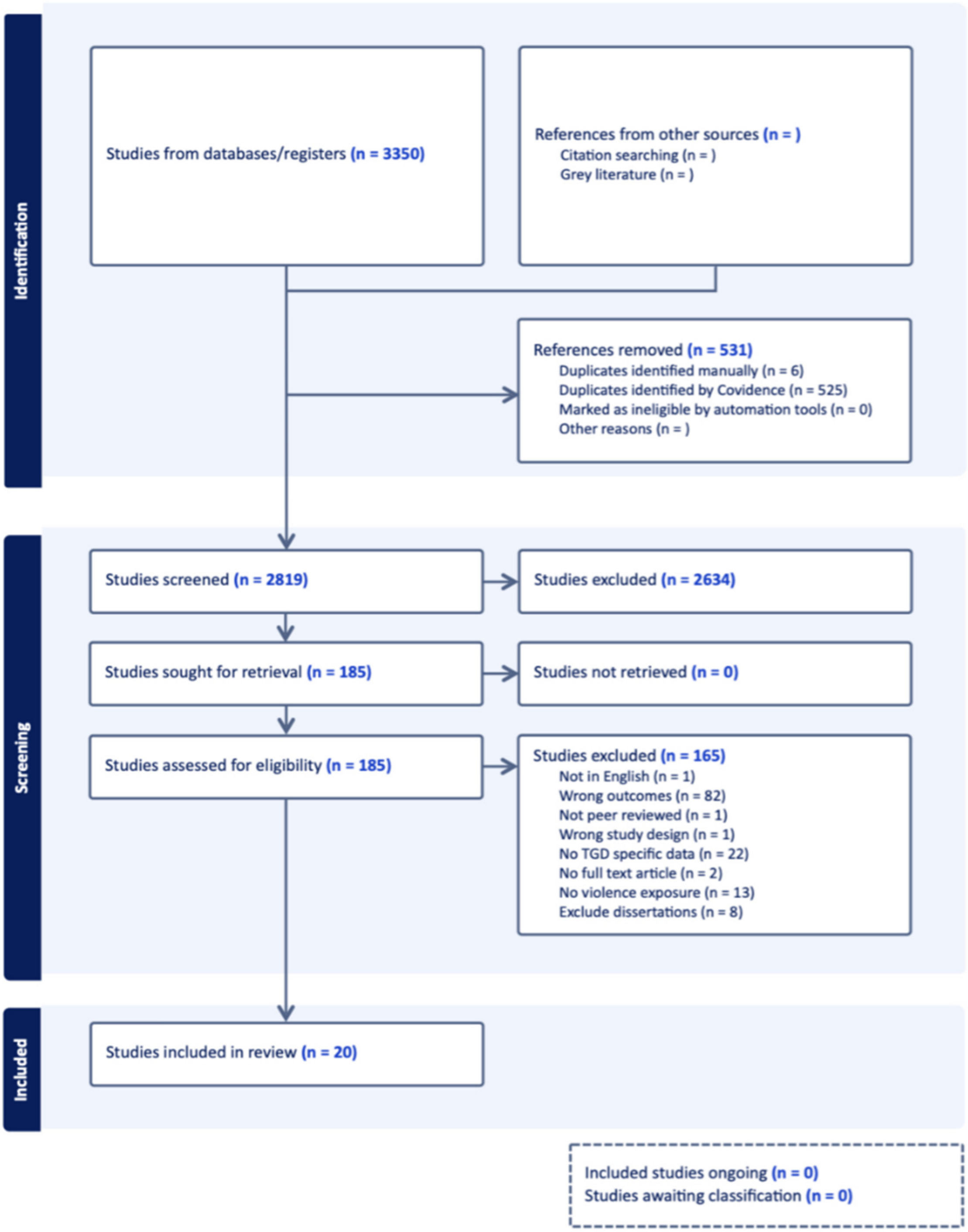

Figure S1. The Preferred Reporting Items for Systematic Reviews and Meta-Analyses flow diagram.

Table S1. Quality Appraisal of Included Articles.

**Included Articles**	**Quality Appraisal**									
Clinical Trials										
Arayasirikul et al. 2022	14/16 CONSORT	**Structured summary…**	**Scientific background & rationale**	**…objectives or hypotheses**	**…trial design…**	**Important changes to methods …**	**Eligibility criteria…**	**Settings and locations …**	**…pre-specified primary and secondary outcome…**

		No	Yes	Yes	Yes	Yes	Yes	Yes	Yes
		**Statistical methods …**	**…periods of recruitment and follow-up**	**A table showing baseline demographic…**	**For each primary and secondary outcome, results…**	**Trial limitations…**	**Generalizability…**	**Interpretation consistent with results…**	**Sources of funding and other support…**
		Yes	No	Yes	Yes	Yes	Yes	Yes	Yes
					Cross-sectional Quantitative Studies				
		**...sample clearly defined?**	**...subjects and the setting…**	**Was the exposure measured …**	**Were objective, standard criteria used…**	**Were confounding…**	**Were strategies to deal with confounding …**	**Were the outcomes measured…**	**Was appropriate statistical analysis…**
Beckman et al. 2018	8/8 JBI	Yes	Yes	Yes	Yes	Yes	Yes	Yes	Yes
Garcia et al. 2025	6/8 BJI	Yes	Yes	Yes	Yes	No	No	Yes	Yes
Grocott et al. 2023	6/8 JBI	No	Yes	Yes	Yes	No	Yes	Yes	Yes
Hughto et al. 2021	8/8 JBI	Yes	Yes	Yes	Yes	Yes	Yes	Yes	Yes
Kilimnik et al. 2023	6/8 JBI	Yes	Yes	Yes	Yes	No	No	Yes	Yes
Lacombe-Duncan et al. 2021	4/8 JBI	No	No	Yes	Yes	No	No	Yes	Yes
Laughney et al. 2025	7/8 JBI	Yes	Yes	Yes	Yes	Yes	No	Yes	Yes
Lindsay et al. 2016	6/8 JBI	Yes	Yes	Yes	Yes	No	No	Yes	Yes
López et al. 2025	5/8 JBI	Yes	No	Yes	Yes	No	No	Yes	Yes
Madzoska et al. 2025	6/8 JBI	Yes	Yes	Yes	No	Yes	Yes	Yes	Yes
McDowell et al. 2019	6/8 JBI	Yes	Yes	Yes	Yes	No	No	Yes	Yes
McMillan et al. 2024	6/8 JBI	Yes	Yes	Yes	Yes	No	No	Yes	Yes
Peitzmeier et al. 2019	8/8 JBI	Yes	Yes	Yes	Yes	Yes	Yes	Yes	Yes
Reisner et al. 2016	8/8 JBI	Yes	Yes	Yes	Yes	Yes	Yes	Yes	Yes

Sherman et al. 2020	6/8 JBI	Yes	Yes	Yes	Yes	No	No	Yes	Yes
Sherman et al. 2021	8/8 JBI	Yes	Yes	Yes	Yes	Yes	Yes	Yes	Yes
Sherman et al. 2022	8/8 JBI	Yes	Yes	Yes	Yes	Yes	Yes	Yes	Yes
Sherman et al. 2024	8/8 JBI	Yes	Yes	Yes	Yes	Yes	Yes	Yes	Yes
Strauss et al. 2020	6/8 JBI	Yes	Yes	Yes	Yes	No	No	Yes	Yes
Strauss et al. 2020	6/8 JBI	Yes	Yes	Yes	Yes	No	No	Yes	Yes
Stults et al. 2023	8/8 JBI	Yes	Yes	Yes	Yes	Yes	Yes	Yes	Yes
Suarez et al. 2021	8/8 JBI	Yes	Yes	Yes	Yes	Yes	Yes	Yes	Yes
Sun et al. 2023	6/8 JBI	Yes	Yes	Yes	Yes	No	No	Yes	Yes
Taber et al. 2023	6/8 JBI	Yes	Yes	Yes	Yes	No	No	Yes	Yes
					Qualitative Studies				
Hawkey et al. 2021	24/25 CRF-V2	**Was the purpose …**	**Was relevant background…**	**… design?**	**Was a theoretical perspective…**	**Methods …**	**…selection described?**	**Was sampling done…**	**Was informed consent…**
		Yes	Yes	Participatory Action Research	Yes	Interviews, photovoice	Yes	Not Addressed	Yes
		**…description of site**	**...description of participants:**	**Role of researcher and relationship…**	**Identification of assumptions…**	**Procedural rigor was used…**	**Data analyses…**	**Findings were consistent…**	**Decision trail…**
		Yes	Yes	Yes	Yes	Yes	Yes	Yes	Yes
		**Process of analyzing the data…**	**Did a meaningful picture... emerge?**	**... evidence of...components of trustworthiness...**	**Conclusions were appropriate…**	**The findings contributed to theory development…**			
		Yes	Yes	Yes, Yes, Yes, Yes	Yes	Yes			

CONSORT: CONSORT for Longitudinal Study; CRF-V2: Critical Review Form - Qualitative Studies (Version 2.0); JBI: Joanna Briggs Institute Critical Appraisal Checklist for Analytical Cross Sectional Studies

SEARCH STRATEGIES with databases, limitations applied, number of results and dates conducted

PubMed Initial search 641 results 03/23/2023; PubMed First_update 137 results 4/24/2024; PubMed Second Update 137 results 03/31/2025

((("transgender persons"[MeSH Terms] OR "transgender*"[Tw] or "trans gender*"[tw] OR "trans man"[tw] or "trans woman"[tw] or transwoman[tw] or transwomen[tw] or “trans women”[tw] or transman[tw] or transmasculine[tw] or transfeminine[tw] or “trans masculine”[tw] or “trans feminine”[tw] or Neutrois[tw] or pangender[tw] or “2 spirit”[tw] or “gender variant*”[tw] or transvest*[tw] or “non gender”[tw] or nongender[tw] or pangender*[tw] or “pan gender*”[tw] or polygender*[tw] or “poly gender*”[tw] or androgene*[tw] or intergender*[tw] or “inter gender*”[tw] or androgyn*[tw] or "gender minority"[Tw] OR "non-binary"[Tw] OR "gender diverse"[Tw] OR "gender expansive"[Tw] OR "TGNC"[Tw] OR "gender non-conforming"[Tw] or "gender nonconforming"[tw] OR "transsexual*"[Tw] or "trans sexual*"[tw] OR "MTF"[Tw] OR "male to female"[Tw] OR "FTM"[Tw] OR "female to male"[Tw] OR "agender"[Tw] OR "transgenre"[Tw] OR "transgenero"[Tw] or "trans genero"[tw] OR "travestit*"[Tw] OR "two spirit*"[Tw] OR "crossdress*"[Tw] or "cross dress*"[tw] OR "TGD"[Tw] OR "genderqueer"[Tw] or "gender queer"[tw] OR "transsexualism"[Tw] OR "transgenderism"[Tw] OR "genderfluid"[Tw] or "gender fluid"[tw] OR "bigender"[Tw] OR "transsexualism"[MeSH Terms])) AND (("suicid*"[Tw] OR "PTSD"[Tw] OR "post-traumatic stress"[Tw] OR "posttraumatic stress"[Tw] OR "post traumatic stress"[Tw] or "Stress Disorders, Post-Traumatic"[Mesh] or PTS[tw] or PTSS[tw] OR "self-injurious thoughts"[Tw] OR "Self-injurious behaviors"[Tw] OR "Self injurious behaviors"[Tw] OR "Self-injury"[Tw] OR "Self injury"[Tw] OR "depress*"[Tw] OR "self-harm"[Tw] OR "self harm"[Tw] OR "cutting"[Tw]))) AND (("violence"[MeSH Terms] OR "victim*"[Tw] OR "domestic abuse"[Tw] OR "violence"[Tw] OR "homicide"[Tw] OR "IPV"[Tw] OR "DV"[Tw] OR "IPH"[Tw] OR "crime-based"[Tw] or Atrocit*[tw] OR "emotional abuse"[Tw] OR "psychological abuse"[Tw] OR "assault*"[Tw] OR "torture*"[Tw] OR "polyvictim*"[Tw] OR "poly-victim*"[Tw] OR "familial abuse"[Tw] OR "abuse*"[Tw] OR "perpetrator"[Tw] OR "sexual assault"[Tw] OR "bullying"[Tw] OR "murder"[Tw] OR "fatality"[Tw] or “hate crime*”[tw]))

Embase initial search 656 results 03/28/2023; Embase First update 180 results 04/24/2024; Embase Second update 148 3/31/2025

1. 'transgender and gender nonbinary'/exp OR 'male to female transgender'/exp OR 'female to male transgender'/exp OR 'gender variance'/exp OR 'androgyny'/exp OR 'cross-dressing'/exp OR 'transgender'/exp OR 'transsexualism':ab,ti OR 'transsexuality':ab,ti OR 'trans gender*':ab,ti OR 'trans man':ab,ti OR 'trans woman:ab,ti ' OR transwoman:ab,ti OR transwomen:ab,ti OR 'trans women:ab,ti ' OR transman:ab,ti OR transmasculine:ab,ti OR transfeminine:ab,ti OR 'trans masculine':ab,ti OR 'trans feminine':ab,ti OR neutrois:ab,ti OR '2 spirit':ab,ti OR 'gender variant*':ab,ti OR transvest*:ab,ti OR 'non gender':ab,ti OR nongender:ab,ti OR pangender*:ab,ti OR 'pan gender*':ab,ti OR polygender*:ab,ti OR 'poly gender*':ab,ti OR androgene*:ab,ti OR intergender*:ab,ti OR 'inter gender*':ab,ti OR androgyn*:ab,ti OR 'gender minority':ab,ti OR 'non-binary':ab,ti OR 'gender diverse':ab,ti OR 'gender expansive':ab,ti OR 'tgnc':ab,ti OR 'gender non-conforming':ab,ti OR 'gender nonconforming':ab,ti OR 'transsexual*':ab,ti OR 'trans sexual*:ab,ti ' OR 'mtf':ab,ti OR 'male to female':ab,ti OR 'ftm':ab,ti OR 'female to male':ab,ti OR 'agender':ab,ti OR 'transgenre':ab,ti OR 'transgenero':ab,ti OR 'trans genero':ab,ti OR 'travestit*':ab,ti OR 'two spirit*':ab,ti OR 'crossdress*':ab,ti OR 'cross dress*':ab,ti OR 'tgd':ab,ti OR 'genderqueer':ab,ti OR 'gender queer':ab,ti OR 'transgenderism':ab,ti OR 'genderfluid':ab,ti OR 'gender fluid':ab,ti OR 'bigender':ab,ti OR 'transsexualism':ab,ti
2. 'suicide'/exp OR 'suicidal behavior'/exp OR 'posttraumatic stress disorder'/exp OR 'automutilation'/exp OR 'depression'/exp or ‘PTSD'/exp OR ‘posttraumatic stress'/exp OR ‘post traumatic stress'/exp or PTS:ab,ti or PTSS:ab,ti OR ‘self-injurious thought*'/exp OR ‘Self injurious behavior*'/exp OR ‘Self injury':ab,ti OR ‘self harm':ab,ti OR ‘cutting':ab,ti
3. 'violence'/exp OR 'victim'/exp OR 'domestic abuse'/exp OR 'domestic violence'/exp OR 'emotional abuse'/exp OR 'assault'/exp OR 'torture'/exp OR 'polyvictimization'/exp OR 'offender'/exp OR 'bullying'/exp OR 'sexual assault'/exp OR 'homicide'/exp OR 'fatality'/exp OR 'victim':ab,ti OR 'domestic abuse':ab,ti OR 'homicide':ab,ti OR 'ipv':ab,ti OR 'dv:ab,ti' OR 'iph':ab,ti OR 'crime-based':ab,ti OR atrocit*:ab,ti OR 'emotional abuse:ab,ti' OR 'psychological abuse':ab,ti OR 'assault*':ab,ti OR 'torture*':ab,ti OR 'polyvictim*':ab,ti OR 'poly victim*':ab,ti OR 'familial abuse':ab,ti OR 'abuse*':ab,ti OR 'perpetrator*':ab,ti OR 'sexual assault*':ab,ti OR 'bullying':ab,ti OR 'murder':ab,ti OR murdered:ab,ti OR 'fatality':ab,ti OR 'hate crime*':ab,ti OR violence:ab,ti

Web of Science Initial search 1049 results 3/27/2023 Used Topic filter from drop down from search menu; Web of Science First Update 244 results 4/24/2024 244 Used Topic filter from drop down from search menu; Web of Science Second Update 188 results 4/1/2025 Used Topic drop down from drop down search menu

"transgender persons" OR "transgender*" or "trans gender*" OR "trans man" or "trans woman" or transwoman or transwomen or "trans women" or transman or transmasculine or transfeminine or "trans masculine" or "trans feminine" or Neutrois or "2 spirit" or "gender variant*" or transvest* or "non gender" or nongender or pangender* or "pan gender*" or polygender* or "poly gender*" or androgene* or intergender* or "inter gender*" or androgyn* or "gender minority" OR "non-binary" OR "gender diverse" OR "gender expansive" OR "TGNC" OR "gender non-conforming" or "gender nonconforming" OR "transsexual*" or "trans sexual*" OR "MTF" OR "male to female" OR "FTM" OR "female to male" OR "agender" OR "transgenre" OR "transgenero" or "trans genero" OR "travestit*" OR "two spirit*" OR "crossdress*" or "cross dress*" OR "TGD" OR "genderqueer" or "gender queer" OR "transsexualism" OR "transgenderism" OR "genderfluid" or "gender fluid" OR "bigender" OR "transsexualism" **AND** "suicid*" OR "PTSD" OR "posttraumatic stress" OR "post traumatic stress" or "Stress Disorders, Post-Traumatic" or PTS or PTSS OR "self-injurious thought*" OR "Self injurious behavior*" OR "Self injury" OR "depress*" OR "self harm" OR "cutting" **AND** "violence" OR "victim*" OR "domestic abuse" OR "homicide" OR "IPV" OR "DV" OR "IPH" OR "crime-based" or Atrocit* OR "emotional abuse" OR "psychological abuse" OR "assault*" OR "torture*" OR "polyvictim*" OR "poly victim*" OR "familial abuse" OR "abuse*" OR "perpetrator*" OR "sexual assault*" OR "bullying" OR "murder" or murdered OR "fatality" or "hate crime*"

APA PsycInfo Intitial Search 1400 results 03/27/2023; APA PsycInfo First update 258 results 4/24/2024; APA PsycInfo Second update 114 results 04/01/2025; Abstract (AB) filter for all searches

AB ("transgender persons" OR "transgender*" or "trans gender*" OR "trans man" or "trans woman" or transwoman or transwomen or "trans women" or transman or transmasculine or transfeminine or "trans masculine" or "trans feminine" or Neutrois or "2 spirit" or "gender variant*" or transvest* or "non gender" or nongender or pangender* or "pan gender*" or polygender* or "poly gender*" or androgene* or intergender* or "inter gender*" or androgyn* or "gender minority" OR "non-binary" OR "gender diverse" OR "gender expansive" OR "TGNC" OR "gender non-conforming" or "gender nonconforming" OR "transsexual*" or "trans sexual*" OR "MTF" OR "male to female" OR "FTM" OR "female to male" OR "agender" OR "transgenre" OR "transgenero" or "trans genero" OR "travestit*" OR "two spirit*" OR "crossdress*" or "cross dress*" OR "TGD" OR "genderqueer" or "gender queer" OR "transsexualism" OR "transgenderism" OR "genderfluid" or "gender fluid" OR "bigender" OR "transsexualism") AND AB ("suicid*" OR "PTSD" OR "posttraumatic stress" OR "post traumatic stress" or "Stress Disorders, Post-Traumatic" or PTS or PTSS OR "self-injurious thought*" OR "Self injurious behavior*" OR "Self injury" OR "depress*" OR "self harm" OR "cutting") AND AB ("violence" OR "victim*" OR "domestic abuse" OR "homicide" OR "IPV" OR "DV" OR "IPH" OR "crime-based" or Atrocit* OR "emotional abuse" OR "psychological abuse" OR "assault*" OR "torture*" OR "polyvictim*" OR "poly victim*" OR "familial abuse" OR "abuse*" OR "perpetrator*" OR "sexual assault*" OR "bullying" OR "murder" or murdered OR "fatality" or "hate crime*")

CINAHL Initial Search 496 results 3/27/2023; CINAHL First Update 82 results 04/24/2024; CINAHL Ultimate Second update 95 results 04/01/2025 (Note that the library upgraded from CINAHL to CINAHL Ultimate)

(MH "Transgender Persons+") OR (MH "Trans Women") OR (MH "Trans Men") or (MH "Transsexuals") or (MH "Androgyny (Psychology)") OR (MH "Gender Transition") or ("transgender*" OR "trans man" or "trans woman" or transwoman or transwomen or "trans women" or transman or transmasculine or transfeminine or "trans masculine" or "trans feminine" or Neutrois or "2 spirit" or "gender variant*" or transvest* or "non gender" or nongender or pangender* or "pan gender*" or polygender* or "poly gender*" or androgene* or intergender* or "inter gender*" or androgyn* or "gender minority" OR "non-binary" OR "gender diverse" OR "gender expansive" OR "TGNC" OR "gender non-conforming" or "gender nonconforming" OR "transsexual*" or "trans sexual*" OR "MTF" OR "male to female" OR "FTM" OR "female to male" OR "agender" OR "transgenre" OR "transgenero" or "trans genero" OR "travestit*" OR "two spirit*" OR "crossdress*" or "cross dress*" OR "TGD" OR "genderqueer" or "gender queer" OR "transsexualism" OR "transgenderism" OR "genderfluid" or "gender fluid" OR "bigender" OR "transsexualism") **- AND -** (MH

"Suicide") OR (MH "Suicide, Attempted") or (MH "Stress Disorders, Post-Traumatic") or (MH "Injuries, Self-Inflicted") or ("suicid*" OR "PTSD" OR "posttraumatic stress" OR "post traumatic stress" or PTS or PTSS OR "self-injurious thought*" OR "Self injurious behavior*" OR "Self injury" OR "depress*" OR "self harm" OR "cutting") **AND** (MH "Violence") OR (MH "Domestic Violence") OR (MH "Gender-Based Violence") or (MH "Victims") OR (MH "Crime Victims") or (MH "Emotional Abuse") or (MH "Assault and Battery") OR (MH "Torture") OR (MH "Sexual Abuse") OR (MH "Bullying") OR (MH "Homicide") or ("violence" OR "victim*" OR "domestic abuse" OR "homicide" OR "IPV" OR "DV" OR "IPH" OR "crime-based" or Atrocit* OR "emotional abuse" OR "psychological abuse" OR "assault*" OR "torture*" OR "polyvictim*" OR "poly victim*" OR "familial abuse" OR "abuse*" OR "perpetrator*" OR "sexual assault*" OR "bullying" OR "murder" or murdered OR "fatality" or "hate crime*")

File S1. PRISMA Checklist.

Section and Topic	Item #	Checklist item	Location where item is reported
**TITLE**			
Title	1	Identify the report as a systematic review.	Pg 1
**ABSTRACT**			
Abstract	2	See the PRISMA 2020 for Abstracts checklist.	Pg 2 (See checklist at bottom of document)
**INTRODUCTION**			
Rationale	3	Describe the rationale for the review in the context of existing knowledge.	Pg 2-3
Objectives	4	Provide an explicit statement of the objective(s) or question(s) the review addresses.	Pg 3
**METHODS**			
Eligibility criteria	5	Specify the inclusion and exclusion criteria for the review and how studies were grouped for the syntheses.	Pg 4
Information sources	6	Specify all databases, registers, websites, organisations, reference lists and other sources searched or consulted to identify studies. Specify the date when each source was last searched or consulted.	Pg 4
Search strategy	7	Present the full search strategies for all databases, registers and websites, including any filters and limits used.	Pg 3 and Search Strategies in Supplemental Materials
Selection process	8	Specify the methods used to decide whether a study met the inclusion criteria of the review, including how many reviewers screened each record and each report retrieved, whether they worked independently, and if applicable, details of automation tools used in the process.	Pg 3-4

Section and Topic	Item #	Checklist item	Location where item is reported
Data collection process	9	Specify the methods used to collect data from reports, including how many reviewers collected data from each report, whether they worked independently, any processes for obtaining or confirming data from study investigators, and if applicable, details of automation tools used in the process.	Pg 4
Data items	10a	List and define all outcomes for which data were sought. Specify whether all results that were compatible with each outcome domain in each study were sought (e.g. for all measures, time points, analyses), and if not, the methods used to decide which results to collect.	Pg 4
	10b	List and define all other variables for which data were sought (e.g. participant and intervention characteristics, funding sources). Describe any assumptions made about any missing or unclear information.	Pg 4
Study risk of bias assessment	11	Specify the methods used to assess risk of bias in the included studies, including details of the tool(s) used, how many reviewers assessed each study and whether they worked independently, and if applicable, details of automation tools used in the process.	Pg 4
Effect measures	12	Specify for each outcome the effect measure(s) (e.g. risk ratio, mean difference) used in the synthesis or presentation of results.	Pg. 12-13
Synthesis methods	13a	Describe the processes used to decide which studies were eligible for each synthesis (e.g. tabulating the study intervention characteristics and comparing against the planned groups for each synthesis (item #5)).	Pg. 4
	13b	Describe any methods required to prepare the data for presentation or synthesis, such as handling of missing summary statistics, or data conversions.	Pg 4
	13c	Describe any methods used to tabulate or visually display results of individual studies and syntheses.	Pg 4
	13d	Describe any methods used to synthesize results and provide a rationale for the choice(s). If meta-analysis was performed, describe the model(s), method(s) to identify the presence and extent of statistical heterogeneity, and software package(s) used.	Pg 4
	13e	Describe any methods used to explore possible causes of heterogeneity among study results (e.g. subgroup analysis, meta-regression).	N/A
	13f	Describe any sensitivity analyses conducted to assess robustness of the synthesized results.	N/A
Reporting bias assessment	14	Describe any methods used to assess risk of bias due to missing results in a synthesis (arising from reporting biases).	Supplemental Materials (Quality Appraisal Table)
Certainty assessment	15	Describe any methods used to assess certainty (or confidence) in the body of evidence for an outcome.	Supplemental Materials (Quality Appraisal

Section and Topic	Item #	Checklist item	Location where item is reported
			Table)
**RESULTS**			
Study selection	16a	Describe the results of the search and selection process, from the number of records identified in the search to the number of studies included in the review, ideally using a flow diagram.	Pg 4, supplemental materials (PRISMA diagram)
	16b	Cite studies that might appear to meet the inclusion criteria, but which were excluded, and explain why they were excluded.	N/A
Study characteristics	17	Cite each included study and present its characteristics.	Pg 4-7
Risk of bias in studies	18	Present assessments of risk of bias for each included study.	Supplemental Materials (Quality Appraisal Table)
Results of individual studies	19	For all outcomes, present, for each study: (a) summary statistics for each group (where appropriate) and (b) an effect estimate and its precision (e.g. confidence/credible interval), ideally using structured tables or plots.	Pg 12-13
Results of syntheses	20a	For each synthesis, briefly summarise the characteristics and risk of bias among contributing studies.	Supplemental Materials (Quality Appraisal Table)
	20b	Present results of all statistical syntheses conducted. If meta-analysis was done, present for each the summary estimate and its precision (e.g. confidence/credible interval) and measures of statistical heterogeneity. If comparing groups, describe the direction of the effect.	N/A
	20c	Present results of all investigations of possible causes of heterogeneity among study results.	Pg 15
	20d	Present results of all sensitivity analyses conducted to assess the robustness of the synthesized results.	N/A
Reporting biases	21	Present assessments of risk of bias due to missing results (arising from reporting biases) for each synthesis assessed.	Pg 16-17, Supplemental Materials (Quality Appraisal

Section and Topic	Item #	Checklist item	Location where item is reported
			Table)
Certainty of evidence	22	Present assessments of certainty (or confidence) in the body of evidence for each outcome assessed.	Pg. 15-16, Supplemental Materials (Quality Appraisal Table)
**DISCUSSION**			
Discussion	23a	Provide a general interpretation of the results in the context of other evidence.	Pg 16
	23b	Discuss any limitations of the evidence included in the review.	Pg 16-17
	23c	Discuss any limitations of the review processes used.	Pg 17
	23d	Discuss implications of the results for practice, policy, and future research.	Pg 16-17
**OTHER INFORMATION**			
Registration and protocol	24a	Provide registration information for the review, including register name and registration number, or state that the review was not registered.	Pg 3, 18
	24b	Indicate where the review protocol can be accessed, or state that a protocol was not prepared.	Pg 3
	24c	Describe and explain any amendments to information provided at registration or in the protocol.	Pg 4
Support	25	Describe sources of financial or non-financial support for the review, and the role of the funders or sponsors in the review.	Pg 18
Competing interests	26	Declare any competing interests of review authors.	Pg 18
Availability of data, code and other materials	27	Report which of the following are publicly available and where they can be found: template data collection forms; data extracted from included studies; data used for all analyses; analytic code; any other materials used in the review.	Pg

From: Page MJ, McKenzie JE, Bossuyt PM, Boutron I, Hoffmann TC, Mulrow CD, et al. The PRISMA 2020 statement: an updated guideline for reporting systematic reviews. BMJ 2021;372:n71. doi: 10.1136/bmj.n71. This work is licensed under CC BY 4.0. To view a copy of this license, visit https://creativecommons.org/licenses/by/4.0/

Abstract Checklist

Section and Topic	Item #	Checklist item	Reported (Yes/No)
**TITLE**			
Title	1	Identify the report as a systematic review.	Yes
**BACKGROUND**			
Objectives	2	Provide an explicit statement of the main objective(s) or question(s) the review addresses.	Yes
**METHODS**			
Eligibility criteria	3	Specify the inclusion and exclusion criteria for the review.	Yes
Information sources	4	Specify the information sources (e.g. databases, registers) used to identify studies and the date when each was last searched.	Yes
Risk of bias	5	Specify the methods used to assess risk of bias in the included studies.	Yes
Synthesis of results	6	Specify the methods used to present and synthesise results.	Yes
**RESULTS**			
Included studies	7	Give the total number of included studies and participants and summarise relevant characteristics of studies.	Yes
Synthesis of results	8	Present results for main outcomes, preferably indicating the number of included studies and participants for each. If meta-analysis was done, report the summary estimate and confidence/credible interval. If comparing groups, indicate the direction of the effect (i.e. which group is favoured).	Yes
**DISCUSSION**			
Limitations of evidence	9	Provide a brief summary of the limitations of the evidence included in the review (e.g. study risk of bias, inconsistency and imprecision).	Yes
Interpretation	10	Provide a general interpretation of the results and important implications.	Yes
**OTHER**			
Funding	11	Specify the primary source of funding for the review.	Yes
Registration	12	Provide the register name and registration number.	Yes

From: Page MJ, McKenzie JE, Bossuyt PM, Boutron I, Hoffmann TC, Mulrow CD, et al. The PRISMA 2020 statement: an updated guideline for reporting systematic reviews. BMJ 2021;372:n71. doi: 10.1136/bmj.n71. This work is licensed under CC BY 4.0. To view a copy of this license, visit https://creativecommons.org/licenses/by/4.0/
